# Protein electrophoretic migration data from custom and commercial gradient gels

**DOI:** 10.1016/j.dib.2016.08.018

**Published:** 2016-08-16

**Authors:** Andrew J. Miller, Brandon Roman, Eric M. Norstrom

**Affiliations:** Department of Biological Sciences, DePaul University, 2325 N. Clifton Ave, Chicago, IL 60614, USA

**Keywords:** Electrophoresis, Gradient, Molecular weight, Polyacrylamide, Protein biochemistry

## Abstract

This paper presents data related to the article “A method for easily customizable gradient gel electrophoresis” (A.J. Miller, B. Roman, E.M. Norstrom, 2016) [1]. Data is presented on the rate of electrophoretic migration of proteins in both hand-poured and commercially acquired acrylamide gradient gels. For each gel, migration of 9 polypeptides of various masses was measured upon completion of gel electrophoresis. Data are presented on the migration of proteins within separate lanes of the same gel as well as migration rates from multiple gels.

**Specifications Table**

**Table:** 

Subject area	*Chemistry, Biology*
More specific subject area	*Protein electrophoresis*
Type of data	*Graph*
How data was acquired	*Gel image acquisition using an Aplegen OmegaLum G gel imager.*
Data format	*Raw*
Experimental factors	*Protein electrophoresis, acrylamide gradient*
Experimental features	*Proteins of known mass were subjected to polyacrylamide gel electrophoresis (PAGE) until the dye front reached the bottom of the gel. The migration of the proteins was measured and compared within-gel and across gels.*
Data source location	*Chicago, Illinois, USA*
Data accessibility	*Data is with this article.*

**Value of the data**

• Method and data provide parameters for customization of gradient gel electrophoresis.
• The data provide a measure of the consistency of protein migration in both hand-poured and commercially sourced gradient gels.
• Graphical representation of gel-to-gel consistency of protein migration is provided for both hand-poured and commercially sourced gradient gels from single gels as well as from multiple gels.

## 1 Data

**Fig. 1:**
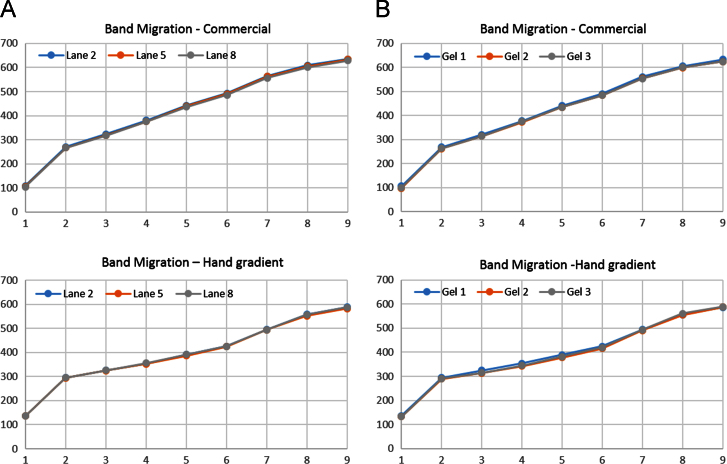
Migration of 9 molecular weight standards measured after electrophoresis on commercial and hand-poured 4–15% acrylamide gradient gels. A). Migration distance was measured in pixels from the stacking gel/resolving gel interface. Data are from a single gel with polypeptides run in lanes 2, 5, and 8. B). Migration distance from the three lanes of each gel was average, and data are presented for each gel.

The data presented here are a graphical representation of the migration of nine polypeptides of known mass after completion of electrophoresis in hand-poured acrylamide gradient gels and commercially sourced acrylamide gradient gels. In Fig. 1A, migration was measured and plotted for multiple lanes within the same gel for both hand-poured and commercial gradient gels. Fig. 1B is a plot of average migration rates of the polypeptides on multiple gels.

## 2 Experimental design, materials and methods

Hand-poured gradient gels were generated using Bio-Rad mini gel casting apparatus. The glass spacer plates consisted of a 1.5 mm spacer, and this was assembled with a short plate creating a resolving gel volume that accommodated 7.5 mL of resolving gel solution. To generate the acrylamide gradient, two solutions were generated in separate vessels. The first consisted 0.375 M Tris–Cl pH 8.8, 0.1% SDS, and 4% acrylamide (29:1 acrylamide:bis-acrylamide). The second solution was identical except that the acrylamide concentration was 15%. To begin polymerization of the acrylamide, 50 µL of a 10% ammonium persulfate solution was added to 10 mL each gel solution followed by 5 µL TEMED. The solutions were separately mixed gently and thoroughly. Using a 10 mL serological pipet and a Drummond Pipet-Aid, 3.5 mL of the 4% acrylamide solution was drawn into the pipet, and this was followed by slowly drawing up an additional 4 mL of the 15% acrylamide solution into the same pipet while avoiding air bubbles. At this point, the pipet contained a total of 7.5 mL of gel solution consisting of two phases of acrylamide density. Next, sufficient air was drawn into the pipet to allow a single air bubble to flow through the 15% acrylamide phase into the 4% phase and to the top of the solution. This causes mixing of the phases at their interface and creates a semi-linear gradient. The solution was then ejected into the assembled casting plates at a rate of approximately 0.25 mL per second. Once all 7.5 mL of solution was ejected, 1 mL of isopropyl alcohol was gently added to the top of the solution and polymerization was allowed to proceed. When polymerization was complete, the isopropyl alcohol was removed and the gel was overlaid with a stacking gel solution consisting of 0.125 M Tris–Cl pH 6.8, 0.1% SDS, and 4% acrylamide along with ammonium persulfate and TEMED. A ten-well comb was inserted into the top of the stacking gel and polymerization was allowed to completion.

Molecular weight markers consisting of nine proteins of approximate masses 250, 98, 64, 50, 36, 30, 16, 10, and 4 kD were run in lanes 2, 5, and 8 of each gel with the other lanes containing Laemmli sample buffer (2% SDS, 10% glycerol, 60 mM Tris–Cl pH 6.8, 0.01% bromophenol blue). The same organization of molecular weight markers was also run on commercially obtained 4–15% gradient gels purchased from Bio-Rad. Electrophoresis was applied at 100 V with constant voltage until the bromophenol blue dye front reached the bottom of the gel at which point the gel was removed from the cassette and imaged using an Aplegen OmegaLum G CCD imager. Using ImageJ software, the images were used to measure the migration of each protein from the top of the resolving gel to its location when electrophoresis was terminated. This measurement was taken in pixel numbers and was plotted as shown in Fig. 1. The data are plotted such that the pixel number is represented on the Y axis and the protein markers are indicated on the X axis where number 1=250 kD, number 2=98 kD and so on. Fig. 1A, top and bottom represent data from a single gel from commercial and hand-poured gels respectively. Next, the migration distance of each marker on three gels that were poured, run, and imaged at separate times was averaged and plotted by gel in Fig. 1B. Discussion on the application and customization of this technique can be found in Ref. [1].
