# Supplementary material for: Protein electrophoretic migration data from custom and commercial gradient gels
Source: Data Brief. 2016 Aug 16;9:1–3. doi: 10.1016/j.dib.2016.08.018 (PMC5007585; doi:10.1016/j.dib.2016.08.018)
Supplement: Supplementary file 1 — Supplementary material [file mmc1.doc]

Department of Biological Sciences

2325 North Clifton Avenue

Chicago, Illinois 60614-3207

773/325-7595

FAX: 773/325-7596


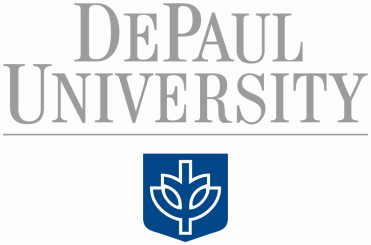


Re: Data in Brief - DIB-D-16-00553

Conflict of interest

The authors declare that they have no conflict of interest with regards to this manuscript.

Sincerely,


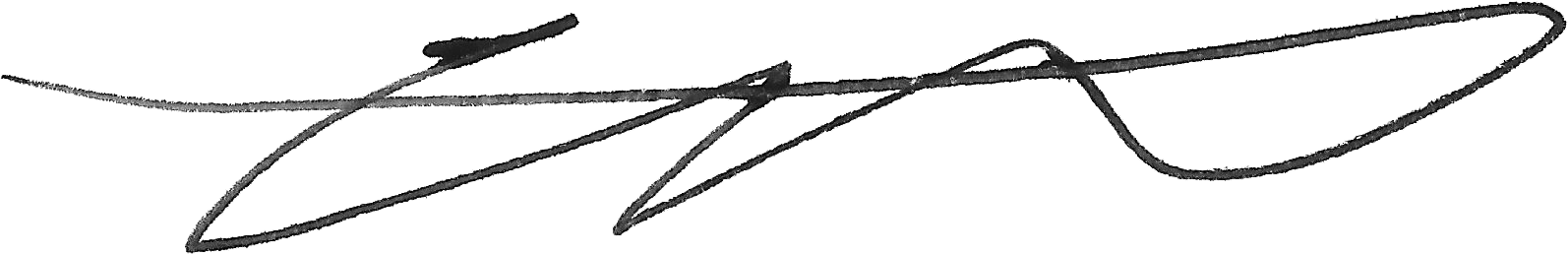


Eric Norstrom, PhD

Assistant Professor

Dept. of Biological Sciences

DePaul University

2325 N. Clifton Ave

Chicago, IL 60614

773-325-2091

[enorstro@depaul.edu](mailto:enorstro@depaul.edu)
